# If you want to save, focus on the forest rather than on trees. The effects of shifts in levels of construal on saving decisions

**DOI:** 10.1371/journal.pone.0178283

**Published:** 2017-05-26

**Authors:** Joanna Rudzinska-Wojciechowska

**Affiliations:** Wroclaw Faculty of Psychology, SWPS University of Social Sciences and Humanities, Wroclaw, Poland; Technion Israel Institute of Technology, ISRAEL

## Abstract

Although financial decisions are expected to be rational, there is a growing body of experimental research indicating that small psychological changes in one’s mind-set in the actual decision-making moment might affect saving ratios. In this article, another type of change in one’s mind-set, which can influence saving decisions, is explored, namely the level of construal. Construal level is a key descriptor of people’s cognitive representations of targets, and is a way of characterising the mental mind-sets people use. Building on recent advances in the link between construal levels and intertemporal choices, the present research evaluates the effect of shifts in levels of construal in the very moment of decision making on people’s propensity to save money. It is suggested that triggering a high-level construal mind-set would influence individuals’ financial decisions and result in greater willingness to save than triggering a low-level construal mind-set. This assumption is supported by the findings: across three experiments, those with an abstract mind-set showed an increased willingness to save when compared to those with a concrete mind-set. The first experiment demonstrated that people in an abstract mind-set are more willing to delay financial gratification than those in a concrete mind-set. In the second and third experiments, those with an abstract mind-set showed an increased willingness to save when compared to those with a concrete mind-set. The research provides further evidence that mental states, which can be evoked by previous, unrelated tasks, such as level of cognitive abstraction, can influence everyday financial decisions. It, thus, highlights the role of situational factors that consumers may be not aware of, which still affect their savings decisions.

## Introduction

The ability to make savings is not only important for national economic systems, it is also essential for the proper functioning of individual households and influences personal well-being. Although, in general, people’s attitudes toward saving are rather positive, their short-term behaviour is often in conflict with their intention to save for the future. As a result, insufficient savings tops the list of adults’ financial worries, as only a fraction of households have enough ‘rainy day’ savings. For instance, in 2013, only 53% of American families declared that they had savings [1]. In the same year, the household net saving rate accounted for 4.9% of household net disposable income in the U.S. and 6.1% in the Euro area [2]. At the same time, the worker-to-beneficiary ratio is falling in many developed countries, which means that social security systems will face severe financial challenges in the near future. Taking this into account, sub-optimal personal savings have become an important social issue. Understanding the mechanisms influencing saving decisions and finding those that encourage and facilitate the ability to put money aside is, therefore, important socially, as well as theoretically.

Although financial decisions, like those concerning saving, are expected to be rational, a wealth of research has shown that the ability to put money aside is not only influenced by economic factors [3–5]. Explanations as to why people fail to save their money focus on variables describing the process of saving itself, such as saving goals, saving motives, saving strategies or saving horizon. They also concentrate on relatively unchangeable traits of a consumer, such as age, the level of education, personality traits and individual differences, for instance level of self-control or time perception. There is also a growing body of experimental research focusing on the actual decision-making moment. Decisions, which are often made once and then are rarely revisited, such as deciding what proportion of a windfall (e.g. heritage, irregular income) should be ascribed into savings or how much of a paycheque to allocate to a pension scheme, might have a significant impact on the level of personal savings. Studies show that variables, which can be described as small psychological shifts in consumers’ mind-set, such as mood [6] fear of death [7], feeling connected with one’s future self [8], feeling powerful [9], or feeling stressed [10], can moderate financial choices. In this article, another type of change in one’s mind-set, which can influence saving decisions, is explored, namely the level of construal.

Construal Level Theory suggests that any action can be construed at varying levels of cognitive abstraction. Events and objects can be represented at either a higher, more abstract level, involving consideration of superordinate goals, desirability, global processing and broad categorisation, or a lower, more concrete level, involving consideration of subordinate goals, feasibility, local processing and narrow categorisations (see [11] for an extensive review of CLT). Research shows that individuals’ judgements, decisions, and behaviors differ as a function of construal levels. Importantly, it has been shown that adopting an abstract construal may result in greater self-control [12]. This also allows people to rise above situational and social influences and, as a result, act in line with their values and beliefs [13]. Moreover, some of the variables that were shown to influence saving decision by altering participants’ mind-set have also been linked, in previous studies, to particular levels of cognitive abstraction. In particular, Guven [6] found that happier people save more, spend less and have a lower marginal propensity to consume, and Cryder et al. [14] demonstrated that sad individuals spend more. Simultaneously, the feeling of happiness was shown to be linked to high levels of construal [15,16]. Similarly, Garbinsky et al. [9] demonstrated that feeling powerful increased saving, and another study, by Smith et al. [17], demonstrated that abstract thinking increases one’s sense of power. Moreover, Hershfield et al. [18] demonstrated that people exposed to their future selves allocate more money into savings (vs. people exposed to their current selves), while Wakslak et al. [19] and Pronin at al. [20] provide evidence that representations of the self at a distant-future time point are more abstract and structured than are representations of the self at a near-future time point. Although these patterns are consistent, the possibility that mere manipulation of the level of construal in the actual decision moment will affect money allocation tasks has not yet received enough attention. The present research evaluates the effect of shifts in levels of construal at the very moment of decision making on people’s propensity to save money. It is hypothesised that high-level construal influences individuals’ financial decisions, and results in greater willingness to save than a low-level construal.

The contributions of the conducted experiments are both theoretical and practical. The research advances the literature on saving and construal level in several ways. It contributes to the financial decision making literature, by proposing another psychological variable that influences saving decisions. It also illuminates the role of situational factors that consumers may be not aware of and yet still affect their financial choices. Furthermore, the experiments add to recent findings in the vast stream of literature on Construal Level Theory by showing another instance in which high-level construal facilitates success at self-control. Perhaps more important are practical contributions of the study, as lack of saving is a significant challenge nowadays. Hopefully, the experiments may ameliorate this challenge, by showing that subtle shifts in one’s perceived level of abstraction in the very moment of financial decision making may affect one’s willingness to save.

## Theoretical background

Consumers, who would like to save money, repeatedly face the same difficult decision. On the one hand, the money they own could be used to gain instant pleasure. On the other, it could be put aside in order to pursue a certain goal in the future. The consequences (positive and negative) of this choice will occur at different points in time. Such decisions are called intertemporal choices [21]. People faced with this kind of decision often weigh immediate outcomes, such as a joy of spending, more heavily than more distant ones, such as accumulating wealth for retirement [22]. As a consequence, despite positive attitudes toward saving and a strong resolution to put money aside, a shift of preferences can be observed and money is spent here and now (see [23] for a review).

According to Construal Level Theory (CLT), changes of preferences often reflect movement on a dimension of psychological distance, a central concept of this framework [24]. Psychological distance changes people’s responses to future events by changing the way people mentally represent those events. It is an important determinant of whether global superordinate primary features of an event or local subordinate secondary features of an event are used while evaluating and decision making. Psychologically distant are all things that are not present in our direct experience, such as other times (past and future), places, the experiences of other people and hypothetical alternative realities, and, as such, they are mental construals [25]. People form high-level construals (more abstract, simple, decontextualised representations) of psychologically distant events and low-level construals (more concrete, contextual representations including incidental features) of proximal events [26]. As a consequence, the value of high-level construals is of higher importance regarding more distant events, whereas the value of low-level construals is of higher importance in the case of more proximal events. Providing that the same event may be construed at either a low or high level of construal, depending on the perceived psychological distance, preferences and decisions might shift as the distance alters. When the value associated with high-level construals is more positive than that associated with low-level construals, the attractiveness of an option should increase with psychological distance and decrease as the distance shrinks [27]. The shift of preferences as a function of psychological distance was presented in such diverse domains as: moral judgements [13], decision making [27,28], impression formation [29] and purchasing decisions [30,31].

The abovementioned shift of preferences can be used to explain sub-optimal, lower than previously planned, saving rates. Like every other decision, a resolution to reduce the level of spending in order to put some money aside involves consideration of the action’s end-state (the final purpose of saving) and means used to reach the end-state (restriction of immediate consumption needs). While having savings is definitely a desirable situation, the necessity of reducing the level of spending is usually seen as unpleasant. According to CLT [26], the value of the action’s end-state reflects a high level of construal of a decision situation since it answers the question why the action is performed. At the same time, means used to reach the end-state reflect a low-level construal of a situation since they provide the details on how the action is to be performed. Hence, saving should seem more desirable from a distance, for example, while planning a monthly or an annual budget since the attention is focused on high-level implications resulting in positive attitudes toward saving. However, when the distance shrinks and it comes to the actual decision whether to save or spend, low level-construals gain importance and saving seems less desirable. It may result in spending money despite previously made resolutions, and a behavior which is inconsistent with previous intentions might be observed.

Construal Level Theory not only explains the abovementioned shifts of preferences and self-control failures but also suggests how to prevent them. Much research in this framework indicates various factors influencing peoples’ levels of representations of a given object or action and, as a result, their self-control levels and possible shifts of preferences. There are individual differences in the tendency to construe the world more or less abstractly [32]. Situational factors that prompt more concrete or abstract thoughts, such as perceived psychological distance to an object or action, also play an important role [25]. However, the psychological distance can be manipulated, for example, by changing perceived spatial [33] or social [34] distance, the probability of an event [35] or its location in time [36]. Extensive research in the framework of CLT shows that there are also effective ways of inducing high and low levels of construals while holding psychological distance constant (see [37] for a review). Such tasks can trigger high- or low-level construals that influence the processing of a subsequent target task.

Taking all this into account, it is hypothesised that shifts in levels of construal might affect people’s propensity to save. Since high-level representations place greater weight on valued goals, people who want to save money may be more able to resist the temptation of immediate spending and decide to save more readily when construing events in high-level terms than when doing so in low-level terms.

**Hypothesis: Triggering a high-level construal mind-set would influence financial decisions and result in greater willingness to save than triggering a low-level construal mind-set.**

The abovementioned hypothesis in line with some previous work focusing on the role of construal level in intertemporal choices. Research in this field has long acknowledged that people’s construal level may affect their level of impatience. In particular, Malkoc et al. [38] demonstrated that abstract processing influences the degree of present bias, i.e the tendency to decrease in required premiums as the delay of consumption gets longer. In the experiments, participants were asked to specify how much compensation they would require to delay the date of receiving goods or benefits. Results showed that high levels of construal lead present-biased preferences to attenuate. Similar results were obtained by Fujita et al. [12]. In this case, participants were asked to indicate how much they would pay to receive certain goods, both immediately and delayed in time, and the authors demonstrated that participants in high-level construal group preferred immediate over delayed outcomes less than those in low-level construal group. Other authors [20,39,40] showed that participants in high-level conditions (induced with a wide range of mind-set manipulation methods) were consistently more likely to choose larger, temporally delayed rewards than a smaller, but immediate ones. In these studies, however, participants were asked to make a single choice between two rewards. Although the results of these studies are promising, a single choice does not provide a measure of individuals’ delay discounting and, therefore, replication of the results using a well-established questionnaire allowing for such a measurement would be beneficial.

Another line of research supporting the previously stated hypothesis directly focuses on saving decisions. Firstly, Ülkümen et al. [41], in a series of studies concerning the role of saving goal specificity, demonstrated that the induced level of construal (study 1) as well as the level of construal influenced by the time horizon to the participant’s saving goal (study 4) is an important moderator for predicting anticipated saving success, and that the chronic level of construal moderates the level of anticipated saving success (study 2) and actual savings (study 3). However, the studies do not provide enough information about the influence of an experimentally induced level of construal on participants’ propensity to save. Secondly, the success of Thaler and Benartzi’s [42] saving plan, called Save More Tomorrow (SMarT), seems to offer another insight into the nature of the relationship between psychological distance and saving decisions. This saving plan asked people to precommit to saving future money from pay rises for their retirement. It turned out to become a very effective tool in getting employees to join the pension scheme and increase their annual saving rates. Although the authors do not interpret the plan in terms of Construal Level Theory, it is worth noticing that the fact that the consequences of the decision were more temporally distant probably led people to give more weight to the benefits of saving and less to the costs of this decision, causing participation to increase [22]. Note, however, that encouraging consumers to postpone the moment they start saving might prove to be counterproductive. For that reason, it is worth checking whether the same effect can be obtained with mind-set manipulation not related to temporal distance.

## Current research

### Ethics statement

All experiments in the present research were approved by the Ethics Committee of Psychological Research at the SWPS University of Social Sciences and Humanities, Faculty in Wroclaw. All subjects in Studies 1–3 provided their informed verbal consent to take part in the research prior to the experiment. Written informed consent was not obtained, in order to protect participants’ anonymity. The consent was obtained twice: first, while participants were being invited to take part in the experiment, and, second, after the experimenter provided more detailed information about the experiment (e.g. the goal and estimated length of the study, and the participant’s rights, as stated in the ethical guidelines of the Ethics Committee). Participants only received all of the study materials after they had agreed to take part in the study. Otherwise, they did not participate in the research. The experimenter documented consent by making a note in the research protocol. The Ethics Committee of Psychological Research at the SWPS University of Social Sciences and Humanities, Faculty in Wroclaw approved this procedure.

### Experiment 1

Work on intertemporal choice has demonstrated that people tend to prefer smaller, immediate rewards over larger, delayed ones (e.g. [43]), and often discount the value of the latter. Despite this, it is widely agreed that reduced temporal discounting is critical for saving money for the future [44]. Opting for larger later rewards might be linked to better saving behavior, as, by repeatedly forgoing short-term rewards, a person could inevitably end up saving more over the long run [45]. The present experiment sought to replicate and extend the results of previous studies suggesting that temporal discounting can be overcome by construing the decision at a high-level. The association between the level of construal and individuals’ delay discounting rates was tested. During the experiment, participants’ levels of construal were manipulated, and, subsequently, their valuation of future versus present rewards was assessed. It was expected that more abstract construals increase individuals’ willingness to wait for future rewards, when compared with more concrete ones.

#### Method

Participants. One hundred and fifty undergraduate students volunteered to take part in the study on how people make decisions regarding their future. The sample size was decided by the number of the participants who signed up for the time period during which the study was run (predetermined to be 10 days). Twenty-four participants were excluded and the remaining sample consisted of 126 participants aged 19–41 (*M* = 22.75, *SD* = 4.58); including 79 females, 40 males, and 7 people who did not specify their gender. The exclusion criteria were set prior to the data coding. Firstly, participants were excluded if they had not followed the instructions provided by an experimenter and completed the dependent measure before the manipulation (*n* = 2; exclusion was based on the experimenter’s notes in the research protocol). Secondly, participants were excluded if they had failed to follow the instruction in mind-set manipulation forms (*n* = 1; exclusion was based on the analyses performed by two competent judges). Thirdly, participants were excluded if they had failed to complete questionnaires. In the case of mind-set manipulation forms, only questionnaires which were 100% complete or missed at most one sentence were coded and included in the data set–as a result, twelve participants were excluded from the sample (five in a low-level construal condition, seven in a high-level construal condition). In the case of Monetary Choice Questionnaire, only questionnaires which were 100% complete were coded and included in the data set. As a consequence, seven participants who circled a single trial on the entire questionnaire rather than circling one alternative on each trial were excluded (4 in a low-level construal condition, 3 in a high-level construal condition). Moreover, there were two participants who met multiple exclusion criteria: one participant had not followed instructions in mind-set manipulation form and failed to meet the criterion of completeness of the mind-set manipulation form (low-level construal condition) and one participant had not completed mind-set manipulation task and circled a single trial in Monetary Choice Questionnaire (low-level construal condition).

Construal level manipulation. Upon arrival, participants were randomly assigned to either a concrete (*n* = 58) or an abstract (*n* = 68) mind-set group and their level of construal was manipulated using a variation of a popular why/how procedure [46], proposed by Henderson [47]. Firstly, all participants listed three things they wanted to accomplish. Next, participants in the high-level condition described three reasons why they wanted to accomplish each thing, and participants in the low-level condition described three ways that they could accomplish each thing. Because consideration of why to perform an action generates abstract thoughts, and consideration of how to perform it results in orientation towards concrete details, this task activates a pattern of high- or low-level thought, respectively, that transfers to new targets [46].

Monetary Choice Questionnaire. After completing a construal level manipulation, all participants were presented with a monetary choice questionnaire [48] (for its psychometric validation see also [49]), which assesses delay-discount rates for monetary rewards. The questionnaire consists of 27 items. For each of them the participant makes a choice between a smaller, immediate amount and a larger, delayed amount (for example, “Would you prefer (a) $34 today or (b) $35 in 186 days?”). The items are grouped into three categories of 9, each based on whether the delayed reward is small ($25, $30, $35), medium ($50, $55, $60) or large ($75, $80, $85). In addition, a final, 28th question was added, similar in form, as well as in the amount and the delay involved, which served as a catch trial: “Which would you prefer to receive, $59 now or $21 in 139 days?” [50]. All participants chose the “$59 now” alternative. All study materials were in Polish. Given the average gross income in Poland and the U.S., the amounts in the questionnaire were not changed ($50 is comparable in its real value to 50 Polish zlotys). Participants took as much time as they needed to complete the procedures, and did not obtain any gratification for taking part in the study. The rewards mentioned in the questionnaire were hypothetical.

Following recent recommendations [50], the respondent’s responses were scored by calculating the percentage of choices of the delayed rewards. This relatively easy measure of individual and group differences in discounting has been proven to be equally reliable to Kirby and Marakovic’s [48] procedure but allows for avoiding the exclusion of data from people who chose either all the immediate or all the delayed options. In addition to each individual’s percentage of delayed reward choices for all 27 questions, the percentage of delayed reward choices was calculated for each of the reward magnitudes (small, medium, large) separately.

#### Results and discussion

Manipulation check. As a manipulation check, two judges coded participants’ level of construal on the basis of their responses to “why” versus “how” questions. Accordingly to suggestions of Fujita et al [12], judges followed the procedure: “if a response reflects a way of accomplishing the previously stated goal, code -1; if a response reflects a reason for accomplishing the previously stated goal, code +1; if the response fits neither criterion or is missing, code 0". The scoring of each participant's nine responses was summed up to create an index of the level of construal ranging from -9 to +9 (higher scores indicate more abstract construal). The codings were highly correlated *(r* = 1, *p* < .001) and were averaged together. As expected, participants’ responses to the why-questions (*M* = 8.91, *SD* = 0.27) were more abstract when compared with their responses to the how-questions (*M* = -8.88, *SD* = 0.31), *t*(124) = -340.03, *p* < .001.

Valuation of future versus present rewards. A 2 (mind-set: concrete vs. abstract) x (3) (magnitude of reward: small, medium, large) analysis of variance was employed, with mind-set factor being between participants and magnitude of reward factor being within participants. The dependent variable was the percentage of delayed choices. Mauchly’s test indicated that the assumption of sphericity had been violated (χ2(2) = 18.90, *p* < .001), therefore, degrees of freedom were corrected using Greenhouse-Geisser estimates of sphericity (ε = .875). The interaction of mind-set and magnitude of reward was not significant, *F*(1.75, 216.96) = 0.394, *p* = .647. As predicted, the main effect of mind-set was significant, *F*(1,124) = 4.316, *p* = .04, partial η ^2^ = .034. As expected, participants decided to delay gratification more often in the abstract construal condition (*M* = 48%, *SD* = 2.543) than in the concrete construal condition (*M* = 41%, *SD* = 2.54). The effect of magnitude of reward was also significant: *F*(1.75, 216.96) = 104.826, *p* < .001, partial η ^2^ = .458. Further analysis revealed that individuals’ percentage of choices of the delayed reward on small amount questions differed significantly from their percentages of such choices on medium amount questions (*p* < .001), which, in turn, differed significantly from their percentages of choices on large amount questions (*p* < .001).

**Fig 1:**
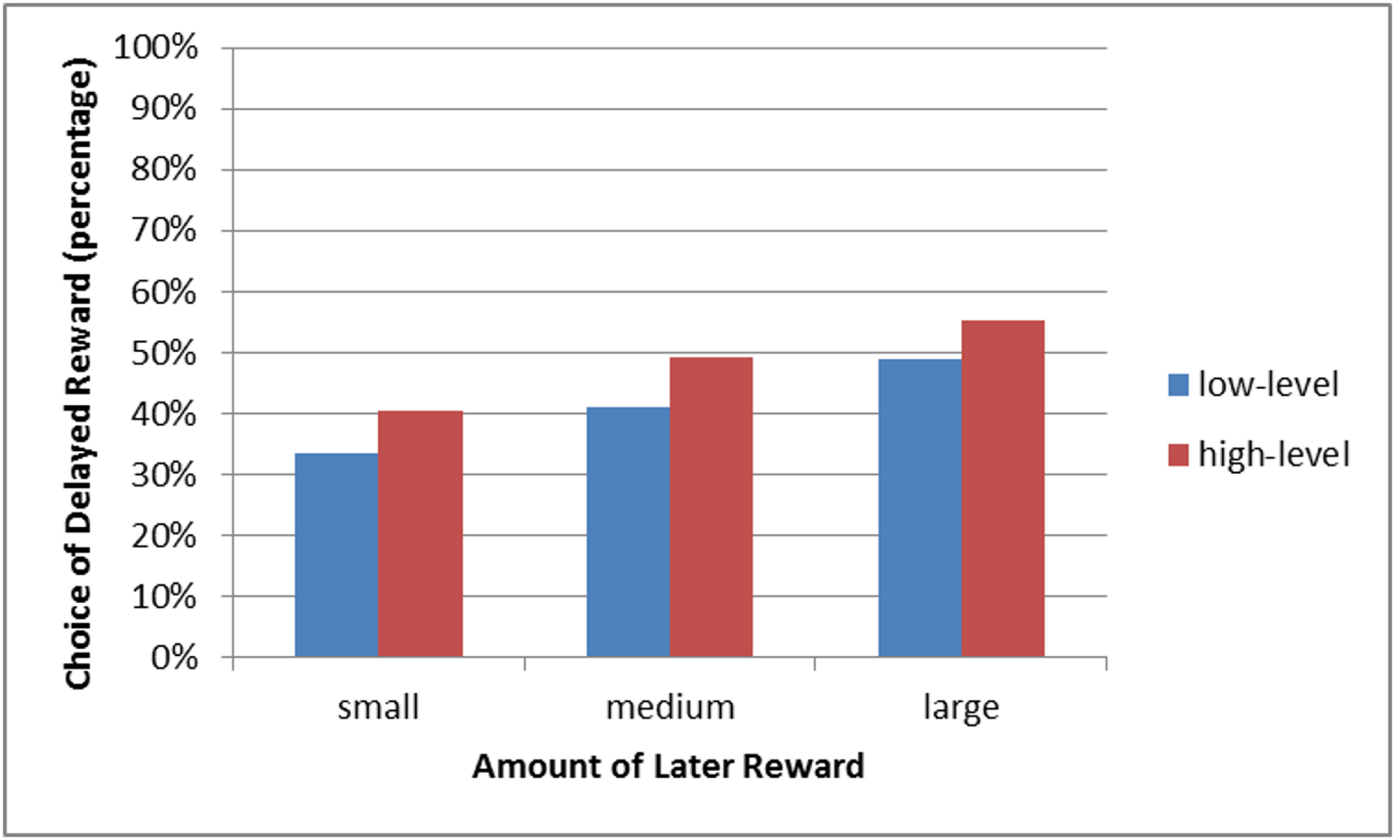
The percentages of participants in low- and high-level conditions choosing the larger, delayed gain on the small, medium, and large amount questions of the Monetary Choice Questionnaire.

Fig 1 reflects the systematic increases in the percentages of choices of the delayed reward as its amount increases from small to medium and, again, from medium to large. These results are consistent with the well-established magnitude effect in delay discounting, that is, the tendency for discount rates to decrease as the magnitudes of the delayed rewards increase [51,52]. As such, they provide evidence that participants’ responses and the method of scoring, based on the proportion of choices of the delayed reward, were reliable.

It can therefore be concluded that, in line with the prediction, inducing high-level construals, compared with inducing low-level construals, led to increased willingness to wait for larger rewards in the future. The ability to delay gratification is key to successful saving, and it is hard to imagine someone devoid of the ability to wait and yet capable of saving. However, does a lower individual discounting rate inevitably leads to greater willingness to save? Note that an increased level of discounting does not only result in the ability to wait for larger rewards. It also involves higher patience, and this may result in lower willingness to pay for instant pleasure or gratification and, thereby lead to more money on hand. What happens with the extra money? It could be saved for the distant future, however, it could also be spent to increase the level of consumption in the moment or in the near future. The question thus arises as to whether the fact that a consumer is unwilling to pay for instant pleasure and doesn’t mind waiting in order to avoid extra fees translates into higher saving rates and willingness to gather long-term savings. Study 2 was designed to seek an answer to this question and, in an attempt to do so, a money allocation task was introduced as a dependent variable.

### Experiment 2

In the second study, participants were asked to complete two ostensibly unrelated questionnaires, which were, in fact, a construal level manipulation and the main task, assessing participants’ financial decisions. Firstly, participants were induced to high- vs. low-level construals using a category versus exemplar task [12]. Secondly, they were asked to divide an unexpectedly received amount of money among various options, ranging from long-term savings to spending on luxuries and pleasure [7]. It was expected that participants in the high-level group would be less willing than participants in the low-level group to allocate money for instant pleasures, and will tend to ascribe more money to long-term savings.

#### Method

Participants. Respondents were 74 Polish university students, who volunteered to participate in two unrelated, consecutive studies, one on how people understand relations between words and categories and one on financial decisions. No compensation was offered. The sample size was decided by the number of the participants who signed up for the time period during which the study was run (predetermined to be 10 days). One participant was excluded from the analysis because her response to the dependent variable question did not add up to 10,000, as it was supposed to. Therefore, the data analysis was conducted on a group of 73 participants (aged 19–33, *M* = 21.11, *SD* = 2.34; 62 females, 5 males, 6 failed to specify their gender).

Construal level manipulation. Construal level was manipulated with a category versus exemplar task [12]. Participants were presented with 40 words (e.g. dog, car, castle). In the abstract-construal condition (*n* = 37), participants generated a superordinate category for each word by answering the question ‘_____ is an example of what?’, whereas in the concrete-construal condition (*n* = 36), they generated a subordinate exemplar for each word by answering the question ‘An example of ______ is what?’ The construal level induced by this task has been shown to carry over to subsequent tasks, and, thus, construal is manipulated without altering information about the prediction target.

Saving/spending task. Participants were asked to imagine that they got a windfall of 10,000 Polish zlotys (approximately €2,500) and had to divide this amount between four options: (1) long-term savings; (2) immediate-access savings; (3) everyday expenses and (4) luxury, pleasurable consumption and immediate dreams fulfilment [7].

An additional study was run among 35 adults (24 women, 11 men), aged 23–40 (*M* = 30.54, *SD* = 4.08) in order to check how the abovementioned options are perceived in terms of spending horizon and to verify whether the last option (involving dreams realization) is perceived as a short-term option, as it was intended by the author. The participants were asked to imagine that someone receives a windfall of 10 000 Polish zlotys and divides it between the four options. Their task was to indicate when–in their opinion–the money assigned to each category will be spent by the person. A seven-point scale (1 –the money will be spent instantly; 7 –the money will be saved for a distant future) was used.

The results of the study confirmed that the categories were understood as it was intended. A one-way repeated measures Anova was conducted to compare participants’ time estimations. Mauchly’s test indicated that the assumption of sphericity had been violated (χ2(5) = 24.54, *p* < .001), therefore, degrees of freedom were corrected using Greenhouse-Geisser estimates of sphericity (ε = .658). There was a significant effect of the allocation option: *F*(1.98, 67.17) = 121.84, *p* < .001, partial η ^2^ = .782; and participants perceived the period before one spends money allocated to:

- long-term savings (*M* = 6.54) as longer than the period before one spends money allocated to any other category, *p* < .001,
- immediate-access savings (*M* = 3.89) as longer than the period before one spends money allocated to everyday expenses (*M* = 1.69), *p* < .001, or to pleasurable consumption (*M* = 2.83), *p* = .023,
- pleasurable consumption as longer than the period before one spends money allocated to everyday expenses (*p* = .01). The last result can be explained given that booking a luxurious trip or buying a watch of one’s dreams takes some time and consideration, definitely more than running everyday errands.

#### Results and discussion

Manipulation check. A similar procedure was employed as in the Experiment 1 [12]. Responses were coded as follows: If the response fit the criterion “[participant’s response] is an example of [target word],” judges coded the response with a score -1. If a response fit the criterion “[target word] is an example of [participant’s response],” judges coded the response with a score 1. If a participant’s response fitted neither criterion or was missing, the response was coded as 0. An index of abstractness (a range from -40 to +40, higher scores indicating a higher construal level) was created by summing up all 40 items. The codings done by the two judges were highly correlated (*r* = 1, *p* < .001) and were averaged together. As predicted, participants who generated category labels (*M* = 39.81, *SD* = 1.16) had significantly more abstract responses than those who generated category examples (*M* = -39.31, *SD* = 0.57), *t*(50.64) = -368.44, *p* < .001.

**Fig 2:**
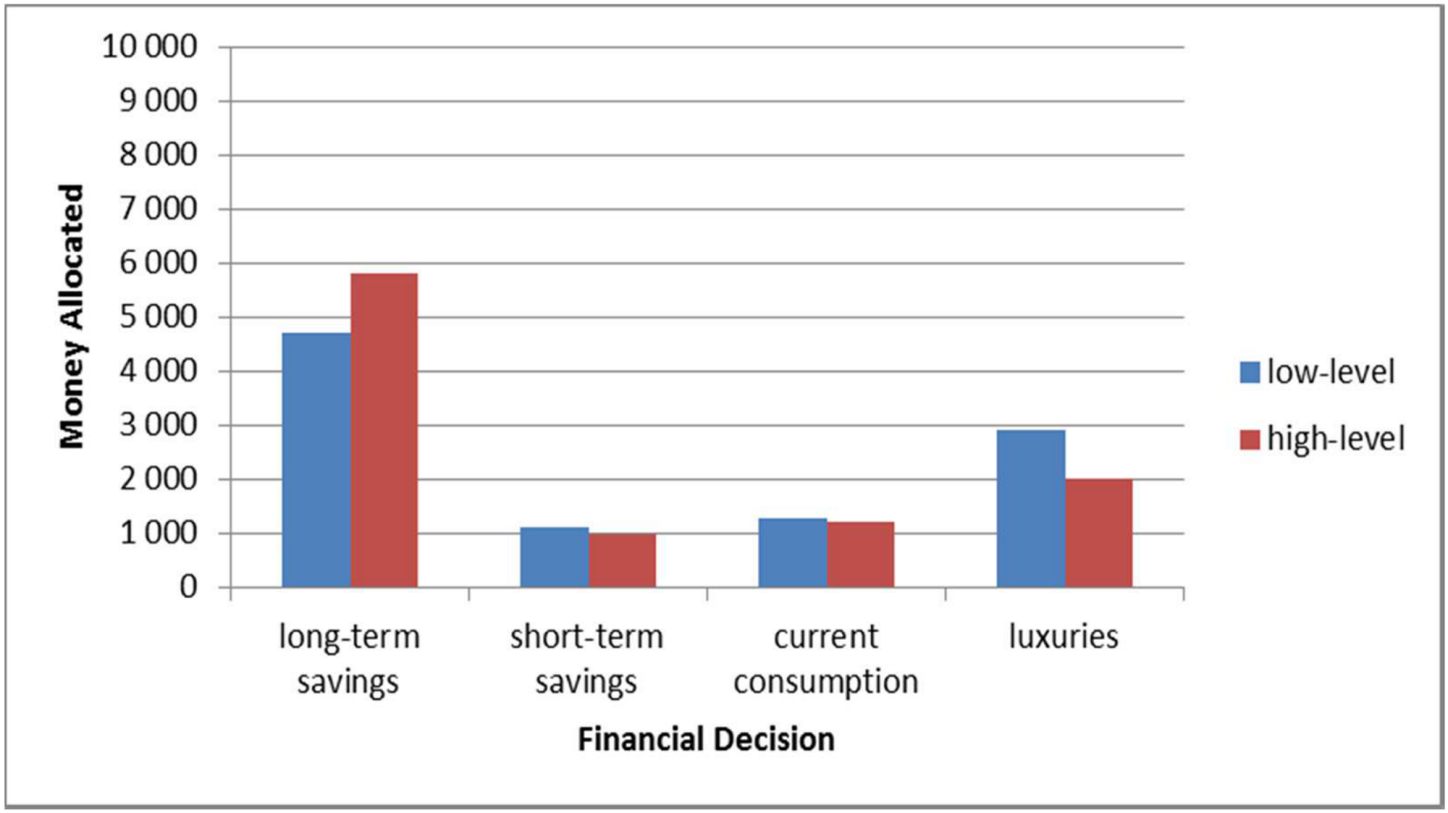
The amount of money allocated into financial categories by participants in concrete and abstract mind-set.

Financial decisions. The financial decisions were analysed with a 2 (mind-set: concrete vs. abstract) x (4) (financial decision: long-term savings, immediate-access savings, everyday expenses, luxury consumption) analysis of variance, with mind-set factor being between participants, and financial decision being within participants. The dependent variable was the amount of money participants decided to allocate. Mauchly’s test indicated that the assumption of sphericity had been violated (χ2(5) = 114.79, *p* < .001), therefore, degrees of freedom were corrected using Greenhouse-Geisser estimates of sphericity (ε = .553). As hypothesised and as can be seen in Fig 2, I found a significant interaction between mind-set and financial decision: *F*(1.66,117.89) = 3.37, *p* = .047, partial η ^2^ = .045. When participants were in a concrete mind-set, they allocated more money to luxurious consumption (*M* = 2919.44, *SD* = 285.99) than people in an abstract mind-set (*M* = 2008.11, *SD* = 282.1), p = .026, as well as allocated less money to long-term savings (*M* = 4708.56, *SD* = 408.09) than people in an abstract mind-set (*M* = 5797.3, *SD* = 402.54), although this effect was only marginally significant, *p* = .061. The effect of financial decision was also significant, *F*(1.66, 117.89) = 74.45, *p* < .001, as participants allocated more money into long-term savings (*M* = 5251.43) than into any other category, *p* < .001, allocated less into short-term savings (*M* = 1036.49) than into long-term savings and luxuries *p* < .001, and less into current consumption (*M* = 1245.53) than into long-term savings and luxuries *p* < .001. The effect of mind-set was not significant, *F* (1,71) = 1.028, *p* = 0.314 (Fig 2).

These findings suggest that people in high-level construal and low-level construal do not differ in their financial decisions regarding consumption of necessities and short-term savings, but make different decisions in the domains of long-term savings and luxury expenses. People in an abstract mind-set tend to allocate more for the future and less on non-necessities in a financial task than people in a concrete mind-set.

### Experiment 3

Whereas Experiments 1 and 2 aimed to establish the relationship between the level of construal and willingness to delay gratification and to engage into long-time savings, this study takes an applied perspective. It aims to check whether the obtained results can be applied in order to intentionally exert a demand or “nudge” in the context of saving decisions. An intervention is proposed, which, in the spirit of using choice architecture to nudge consumers [53], can be used to encourage consumers to increase willingness to save and lower the inclination to excessive current consumption.

It has been shown that influencing one’s mind-set might be successfully applied as an intervention supporting trade-offs between actions that should be done and actions that are desirable. The effectiveness of these kinds of interventions has been demonstrated in various domains, such as promoting reduction of smoking [54], encouraging more positive recycling intentions [55], maximising goal-related behavior in the environmental domain [56] and increasing the adoption of relaxation as a coping behavior [57]. In the domain of personal finances, a construal level intervention was shown to promote a later planned retirement age [58]. In these studies, levels of construal were induced either by tasks unrelated to the subsequent target decision or with a message that activated a given mind-set. They show that construal level interventions are effective in promoting desirability goals over feasibility ones, and their theoretical value is unquestionable. Results of some of them can easily be applied by organisations aiming to promote behaviors that are consistent with attitudes but require some effort, so their practical contribution is also valuable. Nevertheless, it is unrealistic to expect consumers to use these abstract techniques on their own in order to maintain consistency between attitudes and behaviors. What’s more, little is known about strategies that can be consciously used by decision-makers to shift construal levels while choosing between options. Meanwhile, it is important to provide easy techniques for helping to pursue previously set goals, especially in the domain of personal finances and saving. The current study aims to compensate for this limitation. The mind-set manipulation was selected in such a way that it could be used by consumers themselves or by financial advisors to help their clients to stick to their previously set plans and financial goals and to reduce the temptation of excessive consumption. Using the technique could be exceptionally useful while deciding about the amount of pension scheme contributions or what to allocate a lump sum of money for.

The experiment adopted a modification of Freitas, Gollwitzer and Trope’s [46] why/how procedure. It is proposed here, that making participants think about either *why* they save money or *how* they save money might not only be an effective way of inducing respectively high and low levels of construal, but may also serve as an easy self-control technique. In this form, it can be used by consumers themselves in a situation of internal conflict between saving and spending.

#### Method

Participants. Potential participants were approached in two big parks during one summer week and one weekend. They were informed that a survey on saving behavior was being conducted. Next, potential participants were asked whether they try to save money and whether they have their own earnings. Those who answered ‘yes’ to both questions were invited to take part in the survey. After giving their consent, they were asked to fill in open-space questions. The sample size was decided by the number of participants who agreed to take part in the study at the time period when the study was run. A total of 80 participants qualified for participation in the experiment. Due to data missing in one survey, further analyses were conducted using data from 79 participants (46 women, 33 men; aged 19–54, *M* = 37.6, *SD* = 9.06). Participants did not receive any compensation for participation.

Construal level manipulation. Participants were randomly handed one of two versions of the survey, each containing one of two construal mind-set conditions, adapted from Freitas et al. [46]. Those assigned to the abstract construal condition (*n* = 40) were asked to consider why they save money and to list at least three reasons for doing so. Participants assigned to the concrete construal condition (*n* = 39) were asked how they save money and to list at least three ways of putting money aside. Because consideration of why to perform an action generates abstract thoughts, and consideration of how to perform it results in orientation towards concrete details, this task activates a pattern of high- or low-level thought, respectively, that transfers to new targets [46].

Saving/spending task. Participants were asked to imagine that they have just received a certain amount of money they didn’t expect to get and to decide how much money they would save out of it and how much would they spend immediately. Their task was to make four separate decisions referring to the following amounts in Polish zlotys (PLN): 100, 500, 1,000 and 2,000 (approximately €25, €130, €250 and €500). Four different sums of money were introduced, as previous research indicates that people make different decisions when faced with bigger and smaller amounts of money (e.g. [59]).

#### Results

Manipulation check. A manipulation check was performed as in the Experiment 1, but the index ranged from -3 to +3. The codings done by the two judges were highly correlated (*r* = 0.99, *p* < .001) and were averaged together. As predicted, participants who generated reasons for saving money (*M* = 2.86; *SD* = 0.32) had significantly more abstract responses than those who generated ways of saving (*M* = -2.85, *SD* = 0.3), *t*(77) = -81.64, *p* < .001.

The propensity to save. Data were analysed using a 2-way mixed-design ANOVA with a within-subjects factor of the amount of money offered (100 PLN, 500 PLN, 1,000 PLN and 2,000 PLN) and a between-subject factor of construal level (high, low). The dependent variable was the amount of money participants decided to save out of a received sum (converted into a percentage of the sum).

**Fig 3:**
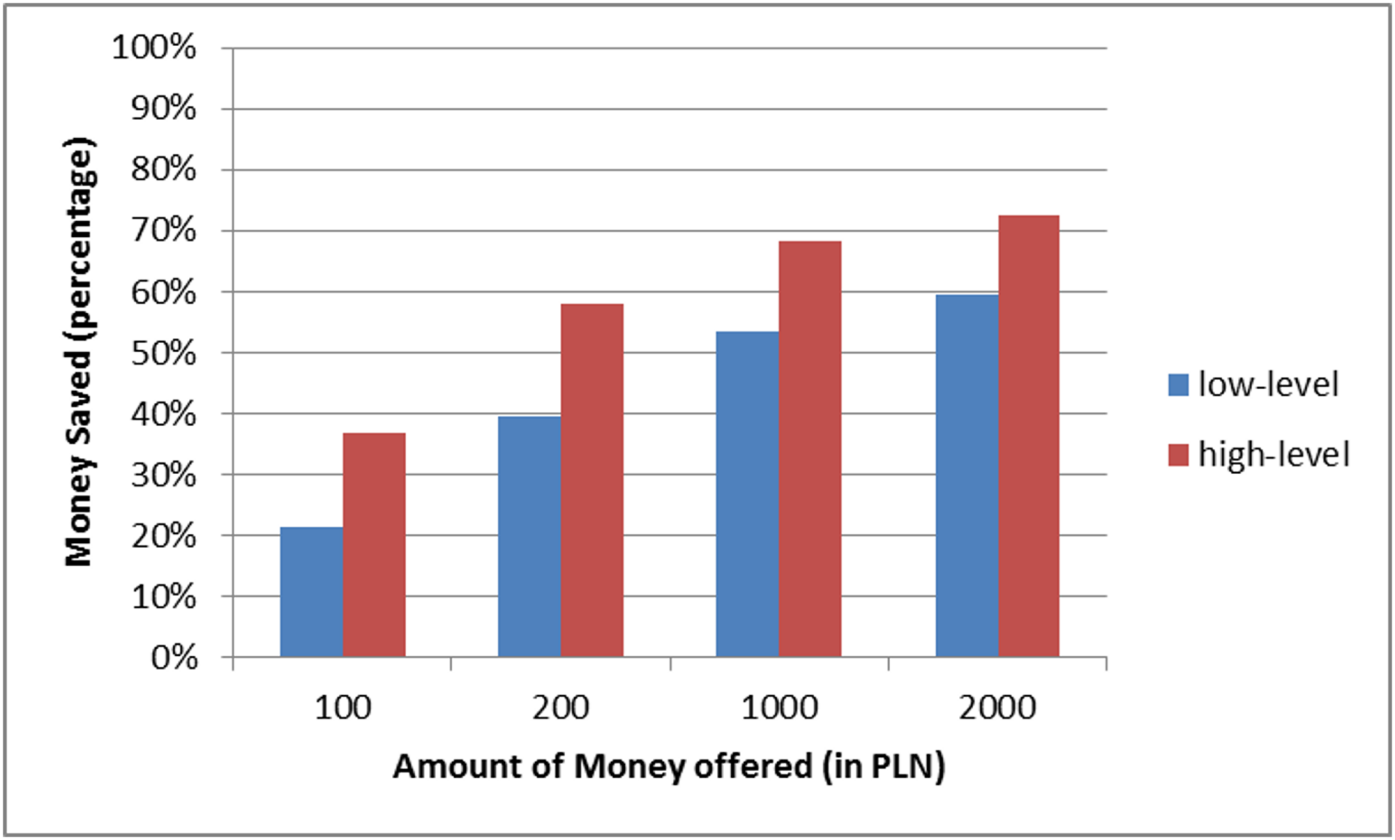
Percentages of offered sums ascribed to saving in high- and low-level construal conditions.

Mauchly’s test indicated that the assumption of sphericity had been violated (*χ*^2^(5) = 82.3, *p* < .001); therefore, degrees of freedom were corrected using Greenhouse-Geisser estimates of sphericity (*ε* = .665). The predicted main effect of construal level proved to be significant: participants in high levels of construal decided to save more money (on average 59% of the offered sum) than participants in low levels of construal (43%, *F*(1, 77) = 10.417, *p* < .01, partial η ^2^ = .119. The main effect of the amount of money offered was also significant: *F*(1.99, 153.54) = 42.198, *p* < .001, partial *η* ^2^ = .354. These main effects were not qualified by an interaction between the amount of money offered and level of construal *F*(1.99, 153.54) = 0.207, *p* = .813, (Fig 3)

Overall, taking into account that there are studies suggesting that low-level of construal is a default mind-set while making financial decisions [38], encouraging consumers who want to save money to focus on the reasons why they want to save seems to be a promising and easy way of helping them to reduce the inclination to excessive consumption and increase their willingness to save.

## Discussion

Convergent findings were obtained in three studies with different measures and manipulations of construal level, using both undergraduate students and community members as participants. The experiments provide evidence that activation of high-level construals in a moment of financial decision-making leads to decreased discounting, lowers the inclination to spend money on excessive consumption and results in higher propensity to save. Moreover, the work also presents a demonstration of an intervention in which people can be encouraged to make more future-oriented choices by having them deliberate on why they want to put money aside.

This work offers novel implications, both theoretical and practical. The results of the experiments advance the literature on saving in several ways. Firstly, they contribute to the vast literature on financial decision-making, by proposing another psychological variable that influences saving decisions. Secondly, the study adds to previous findings, showing that financial decisions are not only based on objective financial data, but are also influenced by individuals’ cognitive representations of them (e.g. [60]). If our decisions reflect subjective construals of events, rather than those events’ objective features [26], the experiments presented above show that financial decisions, and saving in particular, are no exceptions. Furthermore, the findings also extend the growing literature on mind-sets and subsequent choices, which can be influenced due to the carry-over effect. The abovementioned change in cognitive representations might be caused by an activity that the consumer engaged in moments before making a decision, and they may not be aware that this may have an impact on their financial choices. What’s more, the experiments add to recent findings in the vast stream of literature on Construal Level Theory, by showing another instance in which high-level construal facilitates success at self-control. The work also adds to a growing body of literature that examines possible interventions for increasing saving behavior. However, it has to be noted, that the intervention involved hypothetical savings and further studies are necessary in order to assess its effectiveness when real-life choices are made.

Although the obtained results seem promising, the findings and methods have their limitations. Firstly, financial decisions were assessed by relying on quasi-behavioral data obtained in a questionnaire. Therefore, there are no data on actual saving behaviors, rather on saving decisions and intentions. It is demonstrated that activation of high-level construal results in higher willingness to save than activation of a low-level construal. However, whether this it translates into actual saving behavior is a question that needs to be answered in further research.

Nevertheless, the author is aware that conducting a study that might shed some light on the relationship between the level of construal and actual saving behavior might be difficult. On the one hand, it is hard to imagine a research grant enabling researchers to provide participants with incentives high enough to be perceived as worth saving. On the other hand, relying on participant’s own money coming from their regular budget is burdened with such numerous personal variables that the study would be extremely hard to control. Unfortunately, these are common problems that almost all experimental studies attempting to investigate saving decisions share.

Nonetheless, high-level construals are associated with higher self-control, which is essential to saving, and previous studies demonstrated that changes in levels of construal influence real behaviors, not only intentions and decisions [12]. Moreover, although the issue of consistency between saving intention and behavior is severely understudied, there are data indicating that people who plan to save money are usually able to do it. In a study by Rabinovich and Webley [61] more than 80% of Dutch respondents planned to save and were able to realize their plans. The group of people who wanted to save but failed to do so comprised of only 5–6% of respondents. At the same time, data from Belarusian sample show that 37% percent of respondents who wanted to save managed to realize their saving plans and more than 17% failed to do so.

Another limitation of the study is that the rewards in a task used to measure one’s level of discounting were hypothetical. On the other hand, although the use of real rewards is desirable for obvious reasons, there is no clear evidence that the two kinds of rewards are discounted differently [62–64]. What’s more, the temporal duration of mind-set manipulation reminds unknown, as participants made intertemporal choices immediately after experimental manipulation. However, similar to Hershfield et al. [8], the author of this article believes that the most important practical application of such manipulations occurs in a decision-making moment. Finally, it can be argued that the proposed intervention seem to be so effective because it not only activates a high-level of construal, but also reminds participants about their saving goals. By doing so, it might activate goal-related thoughts and behaviors. A possibility needs to be considered that the differences between study conditions led to a difference in meaning the participants assigned to a saving behavior (e.g. it might seem more difficult and less desirable in the low-level condition than in the high-level condition). This is true. However, the goal of the last study was to suggest an intervention aiming to help consumers to refrain from excessive consumption, and, thus, it was only natural to refer to saving and saving goals while trying to evoke high-level construals. The author is aware that the theoretical contribution of the third study is somewhat weaker than that provided by the previous ones. Nevertheless, the effectiveness of the proposed technique and ease of putting it into practice makes it worth spreading the results. Finally, it is worth noticing that all scenarios in the experiments concerned money that is not regular in participants’ monthly budgets. Future research should examine whether people make the same decisions regarding saving when the money comes from their personal monthly budgets. On the other hand, financial decisions concerning lump sums of money, such as windfalls, extra earnings and bonuses, and saving plans or pension schemes contributions seem equally important in gathering money for the future.

### Conclusion

The results of the experiments indicate that focusing on the proverbial forest, rather than on the trees, yields higher propensity to save and that individual’s mind-sets might play an important role in individual’s financial decisions. They also demonstrate that, due to a carry-over effect, factors that are beyond consumer’s control can influence their choices in the financial domain. However, there is some initial evidence that a voluntary change in the mind-set is also possible and it might be used as an easy self-control strategy which can help reduce present consumption in order to gather means for the future.
